# A letter intervention to GP practices to promote prescription uptake in school-age children with asthma during summer holidays (TRAINS study): a pragmatic cluster randomised controlled trial

**DOI:** 10.1038/s41533-025-00475-1

**Published:** 2026-02-10

**Authors:** Rami A Alyami, Rebecca Simpson, Phillip Oliver, Ric Campbell, Steven A Julious

**Affiliations:** 1 Respiratory Therapy Program, College of Nursing and Health Sciences, Jazan University, Jazan, Saudi Arabia; 2 The Sheffield Centre for Health and Related Research, University of Sheffield, Sheffield, UK

**Keywords:** Diseases, Health care, Medical research

## Abstract

In school-aged children, asthma exacerbation rates peak following the return to school after the summer break. A cluster randomised controlled trial (PLEASANT) found that sending a reminder letter from a family doctor to parents of children with asthma during summer holiday led to a 30% increase in prescription collection in August and a decrease in unscheduled care visits after school return in the period September to December. This intervention also resulted in an estimated cost saving of £36.07 per patient per year. We aimed to assess whether informing general practitioner (GP) practices about the PLEASANT trial and its results could lead to its adoptation in routine practice. A pragmatic open label cluster randomised trial was conducted in England, involving GP practices contributing to the Clinical Practice Research Datalink (CPRD). All GP practices in CPRD were stratified by practice size (decile) and randomly allocated (1:1) to either the intervention or control group. In June 2021, the intervention group received a letter from CPRD via mail and email, informing them about the PLEASANT study findings and offering recommendations. The primary outcome was the proportion of children with asthma (aged 4–15) who collected a preventer prescription in August and September 2021. The trial received both University of Sheffield and Independent Scientific Advisory Committee (ISAC) Ethics approval and was registered with ClinicalTrials.gov (NCT05226091). This study included 1389 GP practices and total of 105,746 children with asthma. The practices were randomly assigned to either the intervention group (n = 693 practices, 52,166 individuals) or the control group (n = 695 practices, 53,580 individuals). Analysis showed that 15,716 children (35.3%) in the intervention group and 16,001 children (35.1%) in the control group collected a preventer prescription. No statistically significant difference was found between the two groups (OR 1.01, 95% CI 0.97–1.04), suggesting the intervention had no effect on prescription collection. The study results indicate that a passive intervention, consisting of providing a letter to GPs, did not yield the desired results. To effectively bridge the gap between evidence and practice, it may be worthwhile to consider exploring more proactive strategies to address the identified issues. The trial was registered under ClinicalTrials.gov ID: NCT05226091.

## Introduction

Asthma episodes and deaths have been shown to exhibit seasonal patterns, with several studies indicating peaks in asthma episodes among school-age children upon returning to school following summer vacation^1–5^. When children return to school in the fall, they often come into close contact with allergens and respiratory infections from their classmates^6,7^.

During summer holidays, routines can change and the risks of a respiratory infection might seem lower. This can lead to intentional reduction of preventive asthma medication use or unintentionally forgetting to take it^8,9^. Furthermore, it is been reported that people who are only using bronchodilator therapy, without inhaled steroids or other preventive medications, tend to have higher rates of exacerbation^8,10^.

A prior study confirmed that unscheduled medical contacts with children with asthma increased around the time of returning to school, with children being approximately twice as likely as controls to have an unscheduled medical contact^11^. The same study revealed a 25% drop in inhaled corticosteroid prescriptions in August compared to July and September and that children who did not collect a prescription in August were more likely to have unscheduled care after the return to school.

The factors associated with the decrease in prescriptions in August remain poorly understood. Research on adherence to paediatric asthma treatment has identified weak beliefs about the necessity of asthma medication as a key reason for non-adherence^12^. The decline in asthma medication update during summer months may, therefore, be due to weakened beliefs about the necessity of medicine adherence during a period of perceived lower risk^13^.

The PLEASANT study (Preventing and Lessening Exacerbations of Asthma in School-age children Associated with a New Term)^14^, was a cluster randomised controlled trial involving over 12,000 school-age children across England and Wales. The trial assessed the impact of a simple, doctor-sent letter to parents/guardians emphasising the importance of maintaining regular asthma preventer medication use during the summer holidays. The results showed an 30% increase in prescriptions in August and a subsequent reduction in medical contacts from September to December. This lead to an estimated cost-saving of £36.07 per patient over the year^13^.

The PLEASANT trial showed that a simple, cost-effective intervention could increase preventer prescriptions uptake and reduce unscheduled primary care contacts. There is thus a potential clinical benefit in translating the results of the clinical research from publication to practice. This leads to the motivation for the **TR**ial to **A**ssess **I**mplementation of **N**ew research in a primary care **S**etting (TRAINS) project.

We aimed to assess whether informing General Practitioner (GP) practices of the PLEASANT intervention and study results lead to its adoption. We hypothesised that informing GP practices of the PLEASANT trial findings would increase adoption of the intervention and lead to higher prescription collection among children with asthma in August–September.

## Methods

### Study design

A cluster-randomised controlled parallel-group trial design was employed for the TRAINS study using all GP practices in the CPRD Aurum database in England, as recorded in June 2021. The study included a total of 1389 GP practices. The practices were randomised into two groups: 693 practices in the intervention group and 695 practices in the control group, which continued with their usual care. This design was particularly suitable as it allowed for the use of routinely collected data, thereby ensuring that the study was grounded in real-world, practical conditions^15^.

This trial received ethical approval from the University of Sheffield Research Ethics Committee (Reference Number: 037412). Additionally, an Independent Scientific Advisory Committee (ISAC) approval was granted for Clinical Practice Research Datalink (CPRD) database research (Protocol Reference ID: 21_000436).

### Participants

The target population for the intervention consisted of GP practices in England that were actively contributing to the CPRD Aurum database at that time of the study in June 2021. Data were extracted for school-age children who had been diagnosed with asthma, were registered with an eligible practice and had received preventive prescriptions in the year leading up to the study.

#### Inclusion criteria for practices

- General practices in England that contributed to the CPRD Aurum database on or before June 2021.

#### Exclusion criteria for practices

- General practices located outside of England.
- Practices that left CPRD post-intervention (June 2021) and before the completion of the follow-up o the primary outcome (September 2021).
- Practices that underwent a merger following the intervention and before the end of follow-up period, especially if the merged practices were initially part of different study arms.

#### Inclusion criteria for the data extraction from CPRD

- School-age children, aged between 4 and 16 years old as of 1 September 2021, with a coded diagnosis of asthma, who had been prescribed asthma preventers medication during the past 12 months.

### Randomisation and allocation concealment

GP practices were randomised 1:1 to either intervention or control group. The randomisation process was stratified based on practice size (decile) within the CPRD. The included practices were identified through the CPRD. The process of randomisation was performed by [SAJ] using a bespoke Excel spreadsheet and then reviewed by [RS]. A file with anonymous practice identification codes and the stratification number were provided to the CPRD.

### Procedure

In this study, GP practices enrolled in the CPRD Aurum database and assigned to the intervention group received the intervention package twice—via both email and mail. The intervention package was specifically designed to guide and support GPs, comprising a letter to GPs that highlighted the decline in asthma preventer medication collection during summer holidays, emphasising its significance for children with asthma and presenting findings from the PLEASANT study along with key recommendations and available resources. It also included a leaflet detailing the PLEASANT study, and reminder templates for a SMS text message and GP letter to facilitate adaptation. Further details about the design of the intervention can be found in the protocol^15^. Please see Appendix A for the full the intervention. It is important to note that the letters sent to GP practices were advisory in nature, allowing each practice the flexibility to decide how to practically adopt the intervention as the wished.

The intervention was distributed in June 2021, with emails sent on 23 June and postal packages dispatched on 25 June. This process was coordinated by CPRD. In total, 693 postal packages and emails were sent to GP practices. In some cases, multiple contacts within a practice, such as the practice manager and lead GP, were included, resulting in a total of 1403 email contacts. To monitor engagement, we used CPRD’s email read receipt feature, which enabled us to track whether the CPRD contact lead at each practice opened our email.

Data were extracted from the CPRD Aurum database build in May 2022, focusing on patients aged 4–16 years as of 1 September 2021, who were registered with GP practices in England participating in the TRAINS study. To be eligible, patients needed to have an asthma diagnosis and a prescription for asthma medication within the past year (from 1 June 2020–31 May 2021), indicating active asthma, and must have been alive at the end of the primary analysis period on 30 September 2021.

The extracted records included comprehensive details on all medical contacts, including prescription requests and out-of-hours visits. CPRD provided anonymised data for each patient, which encompassed the General Practice identifier, year and month of birth (for patients under 16), sex, ethnic group, details of prescribed asthma medication, and medical contact data for each appointment, including dates. The research team accessed only fully anonymised data, ensuring that no patient-identifiable information was available.

Following the intervention distribution, data collection continued over a six-month period, ending in December 2021. Two baseline periods, 2019 and 2020, were used for comparison. As 2020 was not considered a standard year due to the impacts of the COVID-19 pandemic we used two years. The primary goal was to evaluate the real-world impact and effectiveness of the intervention during this phase.

### Outcomes

#### Primary outcome

The primary outcome was the proportion of children diagnosed with asthma who collected a prescription for an asthma preventer medication within the time frame of 1st August 2021–30th September 2021.

#### Secondary Outcomes

The following is a list of the secondary outcomes:

1. The total number of prescription collection of asthma preventer medication per patient from 1 August 2021–30 September 2021.
2. The total number of prescription collection of asthma preventer medication per patient in August 2021 and September 2021.
3. The proportion of patients who have collected an asthma preventer medication in August 2021 and September 2021.
4. The total number of prescription collection for asthma preventer medication in the 6 months post-intervention starting 1 July 2021.
5. The proportion of patients with unscheduled medical contacts from September 2021 through December 2021, also segmented by individual months.
6. The total unscheduled medical contacts per patient from September 2021 to December 2021, and the individual months from 1 September 2021–31 December 2021.
7. The proportion of patients with medical contacts (both scheduled and unscheduled) from September 2021 to December 2021, and the individual months from 1 September 2021–31 December 2021
8. The total medical contacts (both unscheduled and scheduled) per patient from September 2021 to December 2021, and the individual months from 1 September 2021–31 December 2021.

### Sample size

The target sample size for this study was determined based on feasibility and the expected number of GP practices providing data to the CPRD. We anticipated including approximately 1389 GP practices (693 in the intervention group and 695 in the control group). Drawing on data from the previous PLEASANT study, we expected around 85 school-age children with asthma per practice^13^. Assuming a 30% prescription collection rate and an intraclass correlation coefficient of 0.03, the study aimed to achieve a precision level estimated as a half-width of 1% for the 95% confidence interval.

This precision-based approach was selected due to feasibility constraints and the primary objective of the TRAINS trial: to accurately estimate the proportion of school-aged children with asthma who collect their prescriptions in each group. Precision-based calculations are particularly suited for trials focused on estimating rates or proportions with high accuracy. Although the study does not have a pre-specified effect size for power calculations, it is designed to provide an accurate estimate of any observed effect.

With an anticipated precision of approximately 1%, the study aimed to deliver a reliable estimate of prescription collection rates in the context of asthma management for school-aged children.

### Statistical analysis

Baseline characteristics of both practices and patients were reported and compared between the treatment groups. At the patient level, descriptive statistics were provided, including gender, age group, and ethnicity (presented as frequencies and percentages), along with age (reported as mean, standard deviation, median, interquartile range, minimum, and maximum). At the practice level, we presented the practice size (deciles), Index of Multiple Deprivation (IMD) (as frequencies and percentages), and the number of children per practice (reported as mean, median, interquartile range, range, standard deviation, minimum, and maximum).

The primary analysis of effectiveness was conducted in the intention-to-treat (ITT) population, which included all practices with data collected during the study period. Sensitivity analyses were performed on a subset of ITT practices identified through email read receipt (RR) confirmations for the intervention practice only.

For the primary outcome, a mixed-effects logistic regression model was used to analyse the proportion of children collecting prescriptions in the primary study period, adjusting for gender, age group, ethnicity, and prescription collection in August–September 2019 and 2020 (included as binary covariates). We initially planned to use 2020 alone as the baseline year; however, because prescribing and consultation patterns in 2020 were disrupted by the COVID-19 pandemic, 2019 was also included as an additional baseline comparator. Binary outcomes were analysed using mixed-effects logistic regression, and count outcomes using mixed-effects negative binomial regression. To account for clustering within practices, random effects were applied based on GP practice ID (decile) and IMD levels. Results from logistic regression are reported as odds ratios (ORs) with 95% confidence intervals (CIs), and results from negative binomial models as incidence rate ratios (IRRs) with 95% CIs. Analyses of unscheduled and total medical contacts across different time periods were performed using the same modelling approach.

All analyses were performed in Stata (version 17), and R (version 4.2.3) was used for generating time-series visualisations. All analyses were conducted using a two-sided significance level of 5%.

## Results

**Fig. 1:**
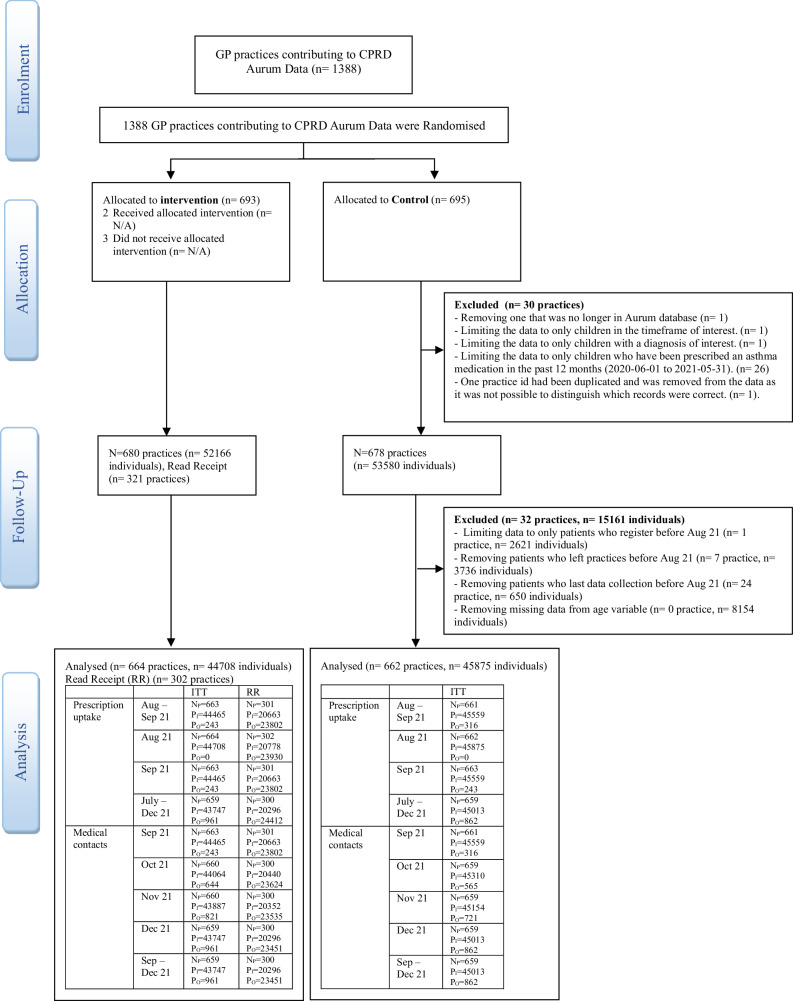
Shows the CONSORT flow diagram. (RR Read receipt (only intervention), NP Number of practices, PI Patients in analysis, and P0 Patient out of analysis compared to Aug 21).

The study was conducted with 1389 general practices sourced from the CPRD Aurum database in England between 1 June 2022, and 31 December 2022. However, after a series of exclusions, the final cohort was 1326 practices encompassing 90,583 individuals. Specifically, 664 practices with 44,708 individuals were allocated to the intervention (letter) group, and 662 practices with 45,875 individuals were allocated in the control group. For each study period, the number of patients and practices were determined based on the dataset specific to that timeframe. The Consolidated Standards of Reporting Trials (CONSORT) flow diagram outlines the number of patients and practices included in the statistical analysis for each time period, along with the reasons for any exclusions as Fig. 1.

Baseline Characteristics show that the intervention (letter) and control groups were comparable in terms of average age, gender distribution, and ethnicity. The mean age of the patients was 10.08 years (SD 3.18), with an age range from 4–15 years. Within the study population, 60% of children with asthma were male and 40% were female. The majority of patients identified as white (61%). There was a slight difference in the distribution of practices and patients. Descriptive statistics for both groups are provided in Table 1.

**Table 1:** Descriptive statistics at the individuals and practices levels.

**(A) At subject level, descriptive statistics of gender, age group and ethnicity (frequencies and percentages) and age (mean, SD, median, IQR, minimum and maximum).**				
**Variable**		**Control (n** **=** **45875)**	**Letter (n** **=** **44708)**	**Total (n** **=** **90583)**
Gender	Male, n (%)	27,600 (60%)	26,785 (60%)	54,385 (60%)
	Female, n (%)	18,275 (40%)	17,925 (40%)	36,200 (40%)
Age	Minimum	4	4	4
	Median (IQR)	10.0 (8.0–13.0)	10.0 (8.0–13.0)	10.0 (8.0–13.0)
	Mean (SD)	10.1 (3.18)	10.1 (3.18)	10.1 (3.18)
	Maximum	15	15	15
Age group	< 5, n (%)	1855 (4%)	1900 (4%)	3755 (4%)
	5–11, n (%)	26,635 (58%)	26,225 (59%)	52,860 (58%)
	> 11, n (%)	17,385 (38%)	16,585 (37%)	33,970 (38%)
Ethnicity	Asian or Asian British, n (%)	5745 (13%)	5700 (13%)	11,445 (13%)
	Black, Black British, Caribbean or African, n (%)	2765 (6.0%)	2350 (5%)	5115 (6%)
	Mixed or multiple ethnic groups, n (%)	2060 (5%)	1830 (4%)	3890 (4%)
	White, n (%)	27630 (60%)	27,455 (61%)	55,085 (61%)
	Other ethnic group, n (%)	860 (2%)	745 (2%)	1605 (2%)
	Not Reported, n (%)	6815 (15%)	6630 (15%)	13,445 (15%)
Baseline: The proportion of children who had a prescription	August-September 2020, n (%)	18,375 (40.1%)	17,792 (39.8%)	36,167 (39.9%)
	August-September 2019, n (%)	15,061 (32.8%)	14,756 (33.0%)	29,817 (32.9%)
**(B) At practice level, descriptive statistics of Practice size deciles, IMD (frequencies and percentages) and number of children per practice (mean, median, IQR, range, SD, minimum and maximum)**				
**Variable**		**Control (n** = **662)**	**Letter (n** = **664)**	**Total (n** = **1326)**
Practice Size (deciles)	1, n (%)	64 (9.7%)	62 (9.3%)	126 (9.5%)
	2, n (%)	65 (9.8%)	65 (9.8%)	130 (9.8%)
	3, n (%)	63 (9.5%)	67 (10.1%)	130 (9.8%)
	4, n (%)	71 (10.7)	69 (10.4%)	140 (10.6%)
	5, n (%)	66 (10.0%)	69 (10.4%)	135 (10.2%)
	6, n (%)	66 (10.0%)	67 (10.1%)	133 (10.0%)
	7, n (%)	67 (10.1%)	65 (9.8%)	132 (10.0%)
	8, n (%)	64 (9.7%)	64 (9.6%)	128 (9.7%)
	9, n (%)	71 (10.7%)	72 (10.8%)	143 (10.8%)
	10, n (%)	65 (9.8%)	64 (9.6%)	129 (9.7%)
IMD	1, n (%)	48 (7.3%)	41 (6.2%)	89 (6.7%)
	2, n (%)	46 (6.9%)	61 (9.2%)	107 (8.1%)
	3, n (%)	59 (8.9%)	49 (7.4%)	108 (8.1%)
	4, n (%)	53 (8.0%)	51 (7.7%)	104 (7.8%)
	5, n (%)	74 (11.2%)	64 (9.6%)	138 (10.4%)
	6, n (%)	72 (10.9%)	69 (10.4%)	141 (10.6%)
	7, n (%)	73 (11.0%)	66 (9.9%)	139 (10.5%)
	8, n (%)	72 (10.9%)	76 (11.4%)	148 (11.2%)
	9, n (%)	74 (11.2%)	94 (14.2%)	168 (12.7%)
	10, n (%)	91 (13.7%)	93 (14.0%)	184 (13.9%)
Number of children per practice	Minimum	1	1	1
	Median (IQR)	57^37–87^	58^37–89^	58^38–88^
	Mean (SD)	69^52^	67^47^	68^49^
	Maximum	620	388	620

The primary outcome result for the proportion of children with asthma collecting a prescription for an asthma preventer medication between 1 August 2021 and 30 September 2021 shows that the adjusted odds ratio (OR) for the intervention group was 1.01 (95% CI: 0.97–1.04). Specifically, in the intervention group—comprising 44,465 patients from 663 practices—15,716 children (35.3%) collected a prescription. In the control group, which included 45,559 patients from 661 practices, 16,001 children (35.1%) collected a prescription (Table 2). This finding suggests that there was no statistically significant difference in the likelihood of children collecting a prescription between the two groups. The ICC for the primary outcome was 0.009.

**Table 2:** Analysis of prescription.

		*Treatment arm (Logistic regression model)*				*Treatment arm (Negative binomial model)*			
	Time period	Intervention Freq (%)	Control Freq (%)	Adjusted Odds ratio	95% CI	Intervention Mean (SD)	Control Mean (SD)	Adjusted Incidence ratio	95% CI
*(A) For all children in the intention-to-treat population*									
*Prescription uptake*	Aug -Sep	15,716 (35.3)	16,001 (35.1)	1.01	0.97–1.05	0.55 (0.95)	0.55 (0.95)	1.01	0.98–1.03
	Aug	8330 (18.6)	8475 (18.5)	1.01	0.96–1.05	0.24 (0.56)	0.24 (0.56)	1.02	0.98–1.05
	Sep	10,972 (24.7)	11,139 (24.5)	1.01	0.97–1.05	0.31 (0.62)	0.31 (0.62)	1.01	0.98–1.04
	Jul –Dec					1.69 (2.45)	1.68 (2.45)	1.00	0.98–1.02
*(B) For children in the read receipt (RR) population*									
*Prescription uptake*	Aug -Sep	7423 (35.9)	16,001 (35.1)	1.02	0.98–1.07	0.56 (0.95)	0.55 (0.93)	1.02	0.98–1.05
	Aug	3924 (18.9)	8475 (18.5)	1.02	0.96–1.08	0.25 (0.57)	0.24 (0.55)	1.03	0.98–1.08
	Sep	5209 (25.2)	11,139 (24.5)	1.02	0.97–1.08	0.32 (0.61)	0.31 (0.61)	1.01	0.97–1.05
	Jul –Dec					1.72 (2.49)	1.68 (2.43)	1.01	0.99–1.04

The proportions and means are descriptive summary statistics. The adjusted Odds ratios and Incidence ratios with the corresponding CIs are from a formal statistical analysis allowing for covariates and the effect of clustering. Analysis of read receipt (RR) confirmations was conducted for the intervention practice only.

**Fig. 2:**
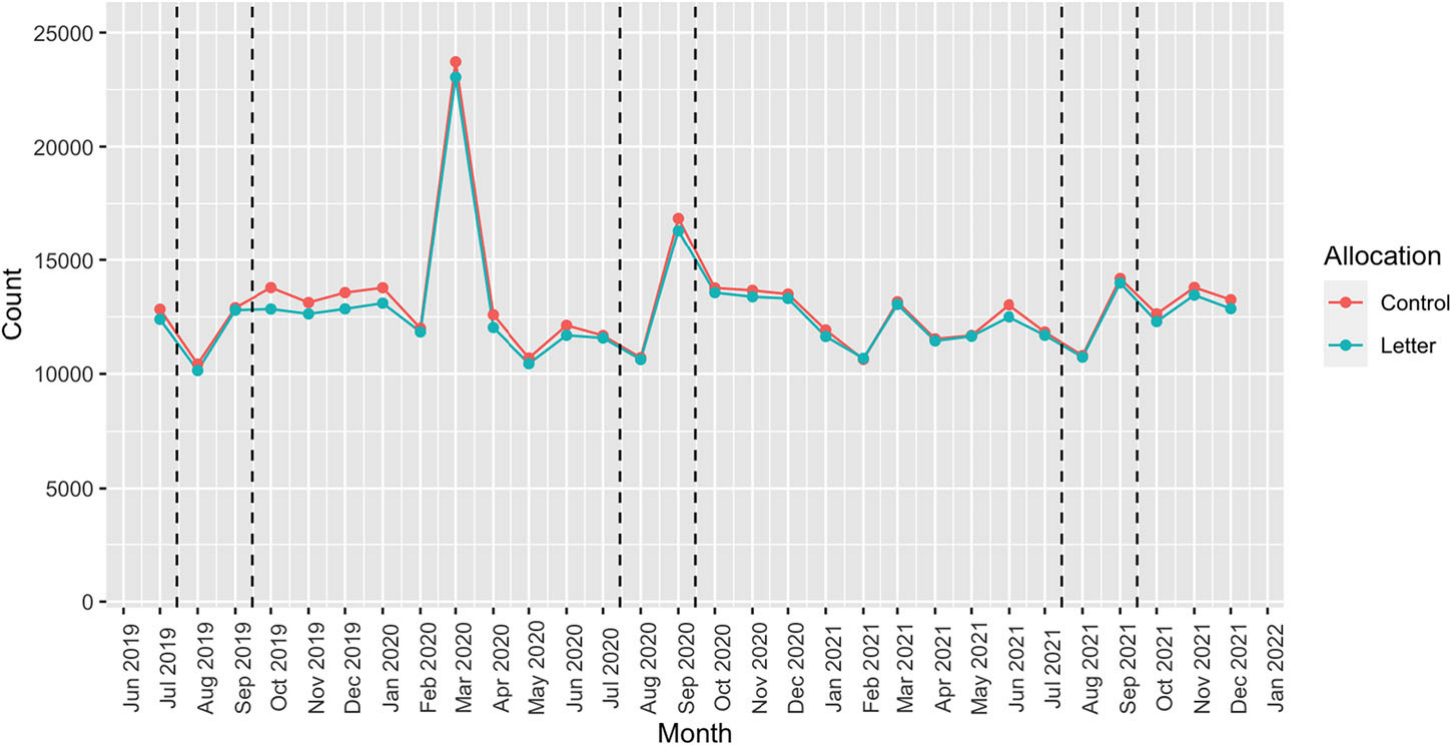
Monthly number of prescriptions picked up by children.

Looking at prescriptions’ secondary outcomes, the OR for the proportion of children who collected a prescription for asthma preventer medication in both August and September were 1.01 (95% CI: 0.96–1.05) and 1.01 (95% CI: 0.97–1.06), respectively. For the number of prescriptions uptakes per patient in August and September 21, the IRR remained consistent at 1.02 (95% CI: 0.98, 1.03) across these periods and remained the same for August, September and July to December 2021 (Table 2). These findings indicate that there was no statistically significant difference in the rates of prescription uptake between the intervention and control groups during the study period. Figure 2 illustrates the monthly prescriptions uptake by children during the study period. It shows a consistent decline observed annually in August.

**Fig. 3:**
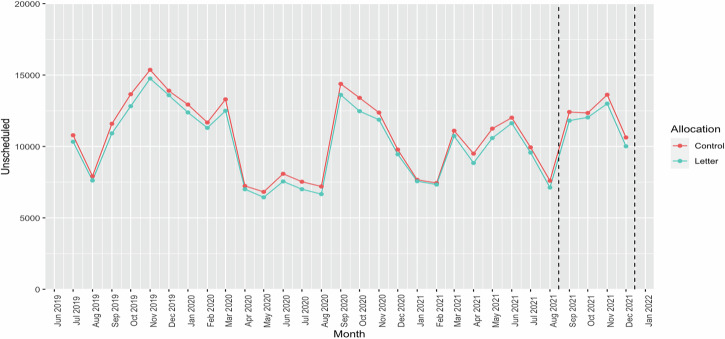
Unscheduled medical contacts by children.

For unscheduled medical contacts analysis across different time periods revealed that the allocation variable did not show statistical significance in any of the five models. Details are provided in Table 3. The analysis of the number of unscheduled medical contacts among children across different time periods is detailed in 3. The results also showed that the allocation variable was not statistically significant. These findings are consistent with the results observed for prescription uptake. Figure 3 shows the number of unscheduled contacts made by the children each month within the study timeframe. It illustrates the consistency and highlights an annual increase in September.

**Table 3:** Analysis of unscheduled and total medical contacts.

		Treatment arm (Logistic regression model)				Treatment arm (Negative binomial model)			
	Time period	Intervention Events (%)	Control Events (%)	Adjusted OR	95% CI	Intervention Mean (SD)	Control Mean (SD)	Adjusted IRR	95% CI
(A) For all children in the intention-to-treat population									
Unscheduled Contacts	Sep	7837 (17.6)	8276 (18.2)	0.98	0.93–1.03	0.24 (0.60)	0.25 (0.60)	0.98	0.94–1.03
	Oct	8066 (18.3)	8.371 (18.5)	0.99	0.94–1.04	0.25 (0.61)	0.25 (0.60)	1.00	0.96–1.05
	Nov	8596 (19.6)	9014 (20.0)	0.97	0.93–1.02	0.27 (0.64)	0.27 (0.64)	0.99	0.95–1.03
	Dec	6878 (15.7)	7286 (16.2)	0.96	0.91–1.01	0.21 (0.56)	0.22 (0.56)	0.96	0.91–1.01
	Sep–Dec	20,369 (46.6)	21,435 (47.6)	0.97	0.92–1.01	0.97 (1.49)	0.98 (1.49)	0.99	0.96–1.02
Total Contacts	Sep	11,190 (25.2)	11,560 (25.4)	1.00	0.95–1.05	0.37 (0.77)	0.38 (0.77)	0.99	0.95–1.03
	Oct	11,678 (26.5)	11,928 (26.3)	1.00	0.96–1.05	0.39 (0.79)	0.38 (0.76)	1.01	0.97–1.05
	Nov	12,195 (27.8)	12,741 (28.2)	0.99	0.94–1.03	0.42 (0.82)	0.42 (0.81)	1.00	0.96–1.03
	Dec	9858 (22.5)	10,345 (23.0)	0.97	0.93–1.02	0.33 (0.72)	0.33 (0.72)	0.98	0.94–1.02
	Sep–Dec	26,653 (60.9)	27,749 (61.6)	0.99	0.94–1.04	1.50 (1.95)	1.51 (1.91)	1.00	0.97–1.02
(B) For children in the read receipt (RR) population									
Unscheduled Contacts	Sep	3734 (18.1)	8276 (18.2)	1.01	0.95–1.08	0.25 (0.60)	0.25 (0.60)	1.02	0.96–1.08
	Oct	3878 (19.0)	8.371 (18.5)	1.02	0.96–1.09	0.26 (0.61)	0.25 (0.60)	1.03	0.97–1.09
	Nov	4082 (20.1)	9014 (20.0)	1.00	0.94–1.07	0.27 (0.63)	0.27 (0.64)	1.00	0.95–1.06
	Dec	3276 (16.1)	7286 (16.2)	0.99	0.93–1.06	0.21 (0.56)	0.22(0.56)	0.98	0.92–1.04
	Sep–Dec	9633 (47.5)	21,435 (47.6)	1.00	0.94–1.06	0.99 (1.49)	0.98 (1.49)	1.01	0.97–1.05
Total Contacts	Sep	5326 (25.8)	11,560 (25.4)	1.03	0.97–1.09	0.38 (0.79)	0.38 (0.77)	1.02	0.97–1.07
	Oct	5536 (27.1)	11,951 (26.4)	1.02	0.96–1.08	0.40 (0.80)	0.38 (0.76)	1.03	0.98–1.08
	Nov	5679 (27.9)	12,759 (28.3)	0.99	0.94–1.05	0.42 (0.83)	0.42 (0.81)	1.00	0.95–1.04
	Dec	4627 (22.8)	10,362 (23.0)	0.98	0.92–1.04	0.33 (0.74)	0.33 (0.72)	0.98	0.93–1.04
	Sep–Dec	12,423 (61.2)	27,749 (61.6)	1.00	0.94–1.06	1.54 (2.00)	1.51 (1.91)	1.01	0.97–1.04

The proportions and means are descriptive summary statistics. The adjusted ORs and IRR with the corresponding CIs are from a formal statistical analysis allowing for covariates and the effect of clustering. Analysis of read receipt (RR) confirmations was conducted for the intervention practice only.

**Fig. 4:**
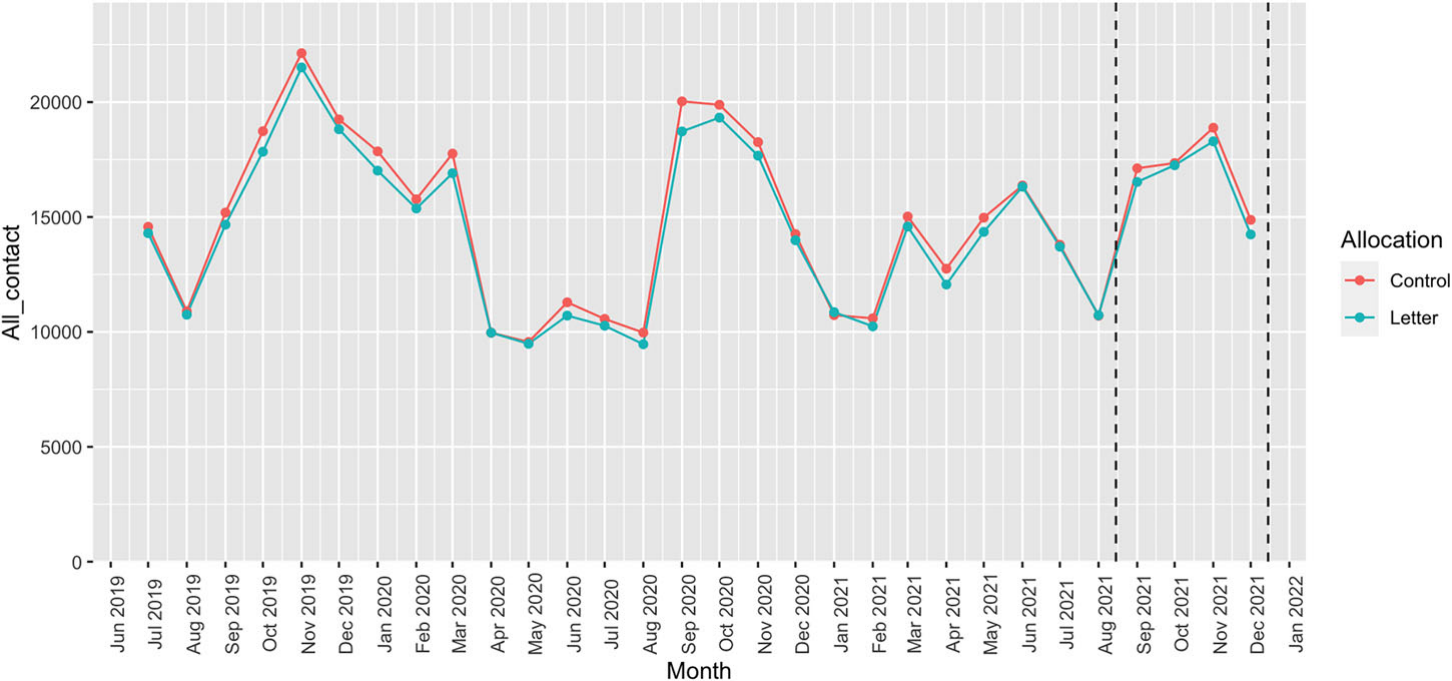
All medical contacts by children.

All medical contacts (both scheduled and unscheduled) results for the proportion of patients with a medical contact showed a consistent OR of 0.99 (95% CI: 0.94, 1.04) from September to December 2021. This ratio remained consistent throughout October, November, December, and the September–December 2021 period, indicating no statistically significant difference in the ORs between the intervention and control groups. The total number of medical contacts per patient was IRR of 1.00 (95% CI: 0.97, 1.02) during this period (Table 3). It indicates no statistically significant difference in incidence rates between the treatments group. This ratio remained relatively constant in October, November, December and the September-December 2021 period, suggesting no statistically significant difference in incidence rates between allocation groups during these periods as well. Figure 4 presented the number of all medical contacts made by the children each month during the study period. The graph shows no difference between the intervention and control arms between September to December 2021, mirroring the patterns from Fig. 3.

A sensitivity analysis was performed using data from the 302 practices (45.5% of the 664 contacted practices) that opened our email in comparison to the control group. The findings were in line with the primary analysis, with an OR of 1.02 (95% CI: 0.98, 1.07) for the proportion of children with asthma who picked up a prescription in August to September 2021 and similar ORs and IRRs for unscheduled and all medical contacts (Table 2, Table 3) indicating no statistically significant difference between the two groups.

## Discussion

To the best of our knowledge, this is the largest cRCT study that has been conducted in the UK, in terms of number of clusters.

Our study’s findings indicate that the passive intervention of sending letters to GPs did not yield the expected results, a finding consistent with a systematic review which showed that printed educational materials (PEMs) have limited impact on GP prescribing behaviours^16^. Similarly, Søndergaard et al.^17^ reported no significant influence of postal feedback on GP prescribing for asthma, even when accompanied by comprehensive clinical data and guidelines.^17^.

However, it is important to highlight the distinctions between previous studies and TRAINS. Earlier studies typically had smaller sample sizes and often used PEMs as control groups, not primary interventions. Additionally, the impact of PEMs on asthma preventer medication prescriptions for school-age children remains unexplored in prior research.

The intervention’s lack of effect may be attributable to several factors including its design and unexpected external challenges like COVID-19. Our reliance on a letter may have been suboptimal, given the mixed results associated with printed educational materials. The process of reaching the GPs was uncertain, and their actions upon receiving the letters could be influenced by constraints such as limited resources and conflicting priorities^18,19^.

Regarding engagement with the intervention, email read receipts indicated that over 45% of practices accessed the emails. However, this did not translate into a noticeable change in their prescribing practices. While there were minor increases in some odds ratios and incidence rate ratios, suggesting a potential trend towards higher prescription rates, these changes were not statistically significant. This underscores the challenges in measuring and interpreting the impact of the intervention.

Moreover, the level of GP compliance in communicating with families remains unclear. Without a mechanism to track this engagement or verify whether the letters were actually delivered to families. This gap in monitoring limits our understanding of how effectively the intervention was adopted and its potential influence on patient outcomes. Additionally, the unexpected COVID-19 pandemic introduced additional challenges, drawing attention and resources away and likely influencing the intervention’s effectiveness^20^.

Even though the intervention did not work, the study also uncovered a recurring annual decline in prescription uptakes during August, and the expected increase in unscheduled care visits from September, were consistent throughout the study, corroborating findings from previous studies^1,4,8,11,13,21^. These patterns align with existing literature and are often attributed to increased exposure to allergens and respiratory viruses upon school resumption^10^.

The COVID-19 pandemic added an unexpected layer to the data, leading to the need for two baselines in the analysis. A significant surge in the prescriptions of asthma preventers in March 2020 was observed, in contrast to the preceding month, February 2020 (Fig. 2). This observable peak corroborates findings reported in other studies^22,23^. Concurrently, the data showed a significant decline in medical contacts, including both all and unscheduled contacts, during the lockdown compared to the previous year (Fig. 4). This trend is also consistent with results found in additional studies^24–26^.

One strength of the study was the use of CPRD for intervention dissemination and data collection, ensuring pragmatic, real-world relevance and comprehensive, continuous data access. The large sample size, inclusion of a diverse patient population, and stratification by practice size deciles bolstered the study’s robustness and generalisability^27^. Furthermore, CPRD enabled continuous, real-time data collection, ensuring data integrity. Lastly, the use of CPRD allowed for the monitoring of the intervention over time, providing valuable insights into the intervention’s long-term impacts and effectiveness.

However, the study has several limitations. The shortages of resources GP practices are in the UK would have lent to the failure of the study as the the study relied on GP practices to have the capacity to send out the intervention. These challenges would be have been amplified by the COVID-19 pandemic.

The pandemic pressures also affected the design, timing and adoption of the intervention, limiting opportunities for in-person seminars with healthcare professionals and shifting GPs’ focus toward urgent patient care, which may have reduced their engagement with the intervention^28,29^. Additionally, the need to add an extra baseline year due to the atypical conditions in 2020 highlights the difficulties in accurately assessing the intervention’s impact. The intervention rollout overlapped with the national COVID-19 vaccination campaign^30^, which likely diverted attention at the practice level. The use of routinely collected data from CPRD posed its own challenges, such as potential missing data, inconsistencies in medical coding, and a lack of standardised definitions, which could affect the accuracy of secondary outcomes. Finally, the absence of a mechanism to confirm whether the intervention reached patients, or if GPs adopted it, made it difficult to fully understand the intervention’s true effectiveness. Overall, these limitations suggest that external factors significantly influenced the study’s outcomes.

Future research could consider shifting the intervention’s focus to higher level GP organisations such Integrated Care Boards (ICBs) or Primary Care Networks for broader impact and exploring the effectiveness of SMS-based interventions and alternative communication methods with GPs as well as interventions in different settings such as schools.

In conclusion, the TRAINS failed to showed that informing GP practices of previous study results had any effect on prescription uptake. Asthma management in school-age children, particularly around their return to school is still important as is the need for any interventions that get research from publication and into practice.

## Data Availability

Access to patient-level data is provided by the CPRD for health research purposes and is dependent on approval of a study protocol by the Research Data Governance (RDG). More information on RDG and the protocol submission process can be found at: https://www.cprd.com/research-applications(date accessed 1 Nov 2024).

## Code availability

All code for data cleaning and processing associated with the current submission is available at 10.15131/shef.data.27291864.
